# Trends in the Exposure, Distribution, and Health Risk Assessment of Perchlorate among Crayfish in the Middle and Lower Reaches of the Yangtze River

**DOI:** 10.3390/foods11152238

**Published:** 2022-07-27

**Authors:** Mengyuan Chen, Manman Wang, Bingjie Zhou, Mengxin Zhou, Qiao Wang, Xin Liu, Yan Liu, Yongning Wu, Xiaole Zhao, Zhiyong Gong

**Affiliations:** 1 Key Laboratory for Deep Processing of Major Grain and Oil (The Chinese Ministry of Education), Hubei Key Laboratory for Processing and Transformation of Agricultural Products, College of Food Science and Engineering, Wuhan Polytechnic University, Wuhan 430023, China; chenmy0126@126.com (M.C.); 15571820885@163.com (M.W.); zhoubj369@163.com (B.Z.); zhoumx1998@126.com (M.Z.); wangqiao8577@163.com (Q.W.); liuxinhook@126.com (X.L.); liuyanwhpu@163.com (Y.L.); wuyongning@cfsa.net.cn (Y.W.); xiaole_1920@163.com (X.Z.); 2 NHC Key Laboratory of Food Safety Risk Assessment, Food Safety Research Unit (2019RU014) of Chinese Academy of Medical Science, China National Center for Food Safety Risk Assessment, Beijing 100021, China

**Keywords:** crayfish, perchlorate, UPLC–MS, risk assessment, inorganic cumulative pollutants, human health

## Abstract

Perchlorate is a well-known thyroid-disrupting chemical as well as an extremely stable inorganic pollutant widely distributed in the environment. Therefore, perchlorate posts potential risks to the environment as well as human health. Crayfish is a dominant aquatic food with increasing consumption levels in recent years. It is crucial to evaluate the accumulation of perchlorate with well-water-soluble properties in crayfish and to assess its health risks. In our present study, we obtained crayfish samples from cultivated ponds and markets based on the regions of the Middle and Lower Reaches of the Yangtze River. The perchlorate concentration was measured in all 206 samples using ultra-high performance liquid chromatography coupled with mass spectrometry (UPLC–MS). Monte Carlo simulation was used to perform health risk assessments. The results indicated that perchlorate levels ranged from 7.74–43.71 μg/kg for cultivated crayfish and 4.90–16.73 μg/kg for crayfish sold in markets. The perchlorate accumulation mainly occurred in exoskeleton parts. All the HQ values were remarkable, at less than one—indicating that perchlorate exposure through the ingestion of crayfish does not pose an appreciable risk to human health.

## 1. Introduction

Perchlorate has become ubiquitous in the environment and can be generated naturally or emitted by human activities, such as through solid rocket fuels, explosives and fireworks, and some fertilizers [1,2,3]. Since the widespread application of perchlorate in industry and agriculture began, a pathway of exposure of organisms, natural water sources, and crops to perchlorate has been provided. Perchlorate can persist for decades and remain mobile in the aquatic environment as it is highly soluble and stable [4], and ultimately becomes concentrated in food that is ingested by humans [5]. The sodium iodide symporter (NIS) is a type of membrane antigen located on the basement membrane of thyroid cells, which plays a pivotal role in the active transport of iodine by the thyroid. However, the affinity of perchlorate to NIS is about 30 times that of iodide ions. Thus, perchlorate can compete with iodide ions for binding to the NIS, which results in interruption of the production of triiodothyronine (T3) and thyroxine (T4) and then causes hypothyroidism [6,7]. In addition, perchlorate can also cause disorders of lipid metabolism in mice [8]. The adverse effects of perchlorate mainly occur in people with thyroid disorders—especially pregnant women and infants [9]. Therefore, perchlorate is putting an increasing strain on public health.

Human exposure to perchlorate occurs primarily through the consumption of water and food [10]. Studies on the dietary exposure risk assessment of perchlorate have been conducted in several regions in recent years. Previous research suggested that food consumption contributed about 80% to the total daily perchlorate intake of humans in Chengdu and Tianjin, China [11]. Dietary exposure to perchlorate is also a subject of considerable interest that has been investigated in many foreign countries and regions. A total of 663 samples have been monitored by Lee to estimate dietary perchlorate exposure in Korea populations, with a 61% detection frequency [12]. Similarly, perchlorate was found in most of fruits and vegetables obtained from retail outlets in Ottawa [13]. Nevertheless, it is important to note that water may be the dominant exposure source of perchlorate for humans due to its water-soluble characteristics [3,14]. In 2017, the limit for perchlorate in drinking water issued by the WHO was 70 μg/L. Therefore, it is also critical to study human exposure to perchlorate in aquatic food. A previous study of perchlorate exposure in shellfish indicated that perchlorate ubiquitously occurred in shellfish from the South China Sea, with a consequential high detection frequency (99.4%) [15]. In addition, perchlorate has been found in other fish as well [3,11].

Crayfish consumption is prevalent in the public, with high nutrition and a delicious taste, and their demand is growing fast [16,17]. Freshwater crayfish is distributed widely in the environment, with a long lifecycle and simple anatomy [18]. Therefore, crayfish could become an excellent indicator of environmental toxic contaminants—especially for water-soluble pollutants. However, information concerning perchlorate contamination in crayfish is limited. China is now the largest producer and consumer of crayfish in the world [16]. According to the development report of the crayfish industry in China in 2021, in the past five years, the cultivation area and output of crayfish in our country have shown continuous growth. In 2021, the crayfish-cultivating area in China reached 26 million mu, with an output of 2.6336 million tons. Therefore, it is crucial to pay attention to contamination in crayfish—especially the water-soluble pollutant perchlorate.

In order to understand the exposure level and distribution of perchlorate in crayfish in the middle and lower reaches of the Yangtze River, in this study, we determined the perchlorate concentration of crayfish based on an established method using ultra-high performance liquid chromatography coupled with mass spectrometry (UPLC–MS). What is more, a health risk assessment of perchlorate exposure in crayfish was evaluated. Owing to the wide range of perchlorate exposure and the limitation of edible portions in crayfish, a probabilistic assessment of perchlorate in crayfish was conducted. Compared with deterministic or semi-probabilistic modelling, probabilistic approaches could capture true variabilities in exposure, such as the variability in perchlorate contamination in crayfish [19]. As a probabilistic assessment method, the Monte Carlo simulation technique has been widely used in food safety risk assessments [20,21,22]. All of these results could provide a reference for the formulation of relevant national standards for perchlorate levels.

## 2. Materials and Methods

### 2.1. Reagents and Materials

Sodium perchlorate standard solution (NaClO_4_, 1000 μg/mL, colorless transparent liquid) and ammonium formate were purchased from Sigma Aldrich (Shanghai, China). The ^18^O-labeled sodium perchlorate standard solution (NaCl^18^O_4_, 100 μg/mL, colorless transparent liquid) was obtained from CIL (Cambridge Isotope Laboratories Inc. Andover, MA, USA). Methanol and acetonitrile at chromatographic grade were purchased from Thermo Fisher (Thermo Fisher Scientific, Waltham, MA, USA). CNW Poly-Sery HLB Pro SPE Extraction cartridges (SBEQ-CA6679-10) were purchased from Anpu Experimental Technology Co., Ltd. (Shanghai, China).

### 2.2. Sample Collection

**Figure 1:**
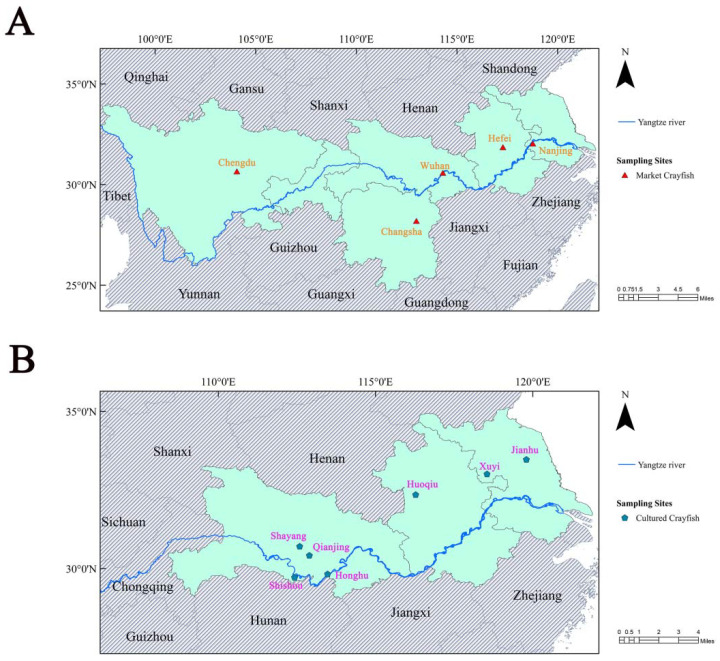
Maps of sampling sites indicating study areas. ((**A**): Sampling sites of market crayfish; (**B**): Sampling sites of cultured crayfish).

The middle and lower reaches of the Yangtze River are the main regions of crayfish cultivation and consumption, where water pollution has received increasing attention [23]. According to the China Crayfish Industry Development Report (2021), Wuhan, Changsha, and 10 other places were selected as sampling sites (Figure 1). To obtain the content and distribution of perchlorate in crayfish from different sources, crayfish for sale in the market (*n* = 14) and pond-cultured crayfish (*n* = 35) were obtained from areas representing the middle and lower reaches of the Yangtze River—where Nanjing, Hefei, Wuhan, Changsha, and Chengdu were the sources of crayfish sold in markets and Xuyi, Huoqiu, Jianhu, Honghu, Shishou, Shayang, and Qianjiang were determined as the sampling origin of cultivated crayfish. For crayfish sold on the market, the systematic sampling strategy was used to randomly collect crayfish samples sold in local supermarkets and aquatic markets, and this sampling method was also adopted for the selection of crayfish culture ponds in every region. Pond-cultured crayfish were obtained directly from local cultivated ponds. A total of 49 kinds of crayfish samples were collected and separated into abdominal muscle, exoskeleton for head and back, hepatopancreas, and other parts. Finally, there were 206 samples acquired for later analysis. All the crayfish samples were stored at −80 °C to avoid corruption.

### 2.3. Sample Extraction and Purification

The samples, including whole crayfish as well as separated tissues, were all thawed at −4 °C conditions. Referring to other pretreatment methods for the determination of perchlorate exposure in meat and fish [3], 2 g wet-weight samples were taken and transferred into 50 mL centrifuge tubes. A total of 13 mL HPLC-grade methanol and 7 mL purified water were added into each tube. Meanwhile, 0.2 mL ^18^O-labeled perchlorate (200 µg/L) was added as an internal standard. Then, vortexing was performed for 2 min. The samples were then centrifuged at 6000 r/min for 10 min. A total of 3 mL HPLC-grade methanol and 3 mL purified water were adopted to rinse the HLB solid-phase extraction column in turn to complete the activation process. A total of 3 mL supernatant of each crayfish sample was transferred into the pre-activated HLB extraction cartridges. Approximately 1 mL filtrate was discarded and the remainder was collected into a 10 mL centrifuge tube. Then, the collected solution was filtered by a 0.22 µm organic filter. Finally, 1 mL solution was added into the vials for UPLC–MS analysis.

### 2.4. UPLC–MS Analysis

The exposure level of perchlorate in each sample was analyzed by ultra-high performance liquid chromatography coupled with mass spectrometry (UPLC–MS; ExionLC AD, Q-Trap 6500+, AB SCIEX, Framingham, MA, USA). The perchlorate was separated on an Acclaim Trinity P1 column (2.1 mm × 100 mm, 3 µm) (Thermo Fisher Scientific, Waltham, MA, USA) in the liquid chromatography system. The chromatography conditions were as follows: mobile phase A and B were 20 mM ammonium formate and acetonitrile, respectively. The gradient procedure flow rate was 0.5 mL/min. The column was maintained at 40 °C. The mobile phase gradient program was set as 30% A and 70% B for the first 2.5 min, then changed to 35% A and 65% B for 2.5 min, and then to 90% A and 10% B for 0.1 min, and then maintained at this ratio for 1.9 min. In the later 0.1 min, the ratio was changed to 30% A and 70% B and maintained for 0.9 min. A 5 µL sample was injected into the column. The auto-sampler temperature was kept at 4 °C.

Mass spectrometry determination was performed with an ESI source in negative mode. Referring to a previous study [3], under the multiple reaction monitoring mode, *m/z* 107 and 89 were monitored for the ^18^O-labeled perchlorate; *m/z* 98.9 and 82.9 and *m/z* 100.9 and 84.9 were monitored for perchlorate to obtain the quantification and confirmation results.

### 2.5. Probabilistic Exposure and Risk Assessment

A probabilistic assessment was implemented to evaluate the perchlorate exposure in crayfish based on the results of perchlorate detection in the abdominal muscle and the hepatopancreas (within the cephalothorax) of crayfish, which are typically consumed by humans. The estimated daily intake (EDI) of perchlorate was calculated according to the following formula [17]:

$$\mathrm{EDI}=\frac{\mathrm{Cs}\times \mathrm{IR}}{\mathrm{Bw}}$$

The Cs is the perchlorate concentration (μg/kg, wet weight) of the studied tissues, and the probabilistic concentrations of perchlorate in the crayfish tissues were calculated by Monte Carlo simulation using the Latin hypercube sampling technique. The probabilistic risk method can avoid the overestimation or underestimation of risks due to the use of deterministic parameters [24]. The simulations were performed with the Microsoft Excel add-in Crystal Ball (@Risk 8.2, Palisade Corp., Raleigh, NC, USA), and 1000 iterations were executed to yield the mean (standard deviation, SD), median, and 95th percentile of dietary intake of perchlorate via crayfish for both adults and children. IR is the ingestion rate—the daily average adult per capita consumption of crayfish based on previous research results (10.54 g capita^−1^ day^−1^, wet weight). Considering the edible parts of crayfish—the abdominal muscle and hepatopancreas—the corresponding food supply quantity of the abdominal muscle (2.11 g capita^−1^ day^−1^, wet weight) and hepatopancreas (1.26 g capita^−1^ day^−1^, wet weight) were calculated based on the mean ratios of abdominal muscle and hepatopancreas to crayfish weight (abdominal muscle/crayfish weight = 20.00 ± 5.01%, hepatopancreas/crayfish weight = 11.96 ± 1.93%, calculated using 430 crayfish with body weights about 30 g). Bw is the body weight, which is 70 kg for adults and 20 kg for children [17].

Then, the hazard quotient (HQ) was calculated based on the following equation to evaluate the health risk of perchlorate exposure in crayfish [16]:

$$\mathrm{HQ}=\frac{\mathrm{EDI}}{\mathrm{RfD}}$$

where THQ > 1 indicates a potential health risk and THQ ≤ 1 reflects that adverse noncarcinogen effects are unlikely and can be considered negligible hazards [25]. The dietary reference dose (RfD) for perchlorate exposure is set at 0.7 μg/kg bw/day, as suggested by US NAS [26].

### 2.6. Quality Assurance and Quality Control

Methodology validation has been conducted to test the analytical technique for the laboratory perchlorate determination. The perchlorate internal standard was used for matrix effect correction. The linearity of the method was determined by six calibration standard solutions (0, 0.25, 0.5, 1, 5, 10, 50 μg/L), with the same internal standard level (2 μg/L). As the obtained result showed, R^2^ > 0.99 indicated a well-fitted calibration curve by regression analysis. Method recovery detection was conducted to evaluate the accuracy. Three different levels of known amounts of certified perchlorate were spiked into the crayfish matrix fluid. The results showed that excellent recoveries were obtained, ranging from 81.05–106.46%. These results suggest that the conventional laboratory method was sensitive and accurate for analyzing perchlorate concentrations in crayfish.

### 2.7. Statistical Analysis

Data analysis was conducted by Microsoft Excel 2016 and SPSS Statistics 25 (IBM SPSS Software, Chicago, IL, USA). All the results were expressed as Mean ± SD. Student’s *t*-tests were performed, and the significance level was set as *p* < 0.05. GraphPad Prism 8 (Graphpad Software, San Diego, CA, USA), Origin 2019b (OriginLab, Northampton, MA, USA), ArcGis software (ESRI Corp., Redlands, CA, USA), and the Microsoft Excel add-in Crystal Ball (@Risk 8.2, Palisade Corp., Raleigh, NC, USA) were used to generate figures.

## 3. Results and Discussion

### 3.1. Detection Frequencies and Concentrations of Perchlorate among Crayfish

To explore the perchlorate level in crayfish, a total of 49 samples—including crayfish cultivated in ponds and sold in markets—were obtained and analyzed by ultra-high performance liquid chromatography coupled with mass spectrometry (UPLC–MS). The occurrence of perchlorate in crayfish is summarized in Table 1. As the results show, overall, perchlorate had accumulated in every sample, with a detection frequency of 100%. The high detection frequency suggested the ubiquitous presence of perchlorate in crayfish obtained from the Middle and Lower Reaches of the Yangtze River. In order to explore changes in the perchlorate content in crayfish during transportation, determination of perchlorate contamination levels in crayfish acquired from two different sources was performed; we found that the accumulation of perchlorate in crayfish sold in markets was evidently lower than that in cultivated crayfish—specifically manifesting as 7.74–43.71 μg/kg in the cultivated crayfish, with a median level of 16.20 μg/kg, and 4.90–16.73 μg/kg in crayfish sold in markets, with a median concentration of 6.15 μg/kg. Previous studies have investigated perchlorate exposure levels in shellfish. For shellfish distributed in the south China sea, they were detected with a median perchlorate level of 4.33 μg/kg, which is lower than that found in our present study [15]. A study on perchlorate exposure levels in various foods from Chengdu showed that the median perchlorate contaminate level in scallops is 5.63 μg/kg, which is also lower than our results here [11]. As for shrimp, their results showed that mantis shrimp were detected with a median level of 13.4 μg/kg and greasy back shrimp were found to have a median level of 4.72 μg/kg [11]. In the present study, Student’s *t*-test was applied to assess differences in perchlorate concentrations between these two sources of crayfish. The results indicated that there was a nearly three-fold difference in perchlorate levels between the two sources of crayfish (*p* < 0.05). The distinct difference in perchlorate levels between the two sources of crayfish suggests that perchlorate metabolism occurred in crayfish during the transport link from the farm to the market.

**Figure 2:**
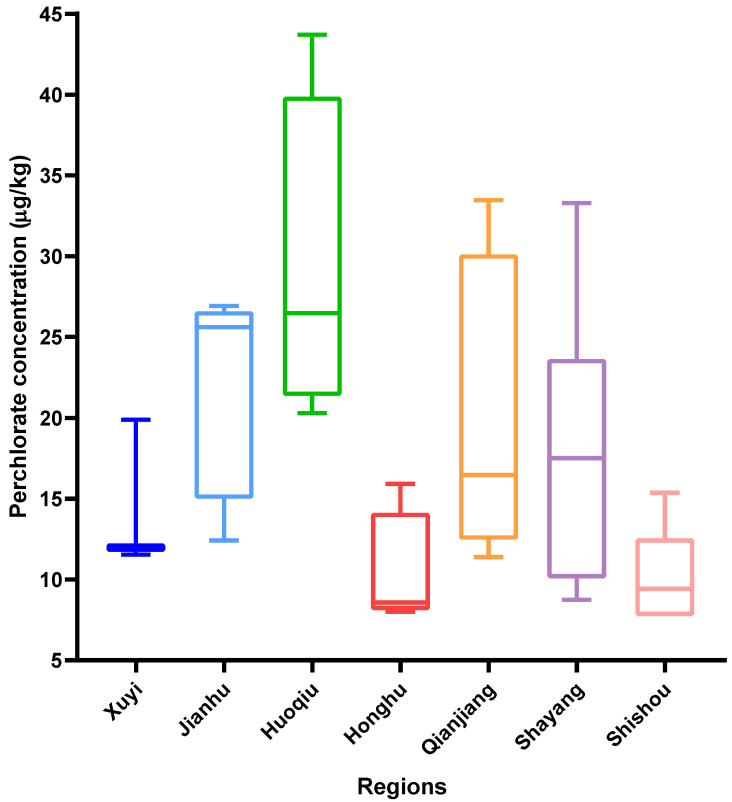
Occurrence of perchlorate in cultivated crayfish among different regions.

Figure 2 presents the exposure to perchlorate in cultivated crayfish among seven different regions in the middle and lower reaches of the Yangtze River. The results indicate that the maximum content occurred in Huoqiu and the minimum was in Honghu, at 29.25 ± 10.19 μg/kg and 10.27 ± 3.78 μg/kg, respectively. It is obvious that the perchlorate concentration of crayfish in Huoqiu was about three times that in Honghu. Student’s *t*-tests were performed to investigate discrepancies in perchlorate contamination levels based on regional differences. Our results indicated that, in general, the exposure level of perchlorate in crayfish of the lower Yangtze regions—including Huoqiu, Jianhu, and Xuyi—was significantly higher than that of the middle Yangtze regions, such as Honghu, Shayang, Shishou, and Qianjiang (*p* < 0.05). Perchlorate can be released into the surrounding environment from industrial production. Due to perchlorate being able to migrate from the environment to the food chain, it will eventually be exposed to organisms and pose a risk to human dietary health. As industrially developed provinces in the middle and lower reaches of the Yangtze River, there are more factories in the region of Jiangsu and Anhui. Therefore, this could be an important factor causing the contamination levels of perchlorate in crayfish in the lower reaches of the Yangtze River to be higher than that in the middle reaches.

### 3.2. Investigation of Perchlorate Concentration in Crayfish Tissues

**Figure 3:**
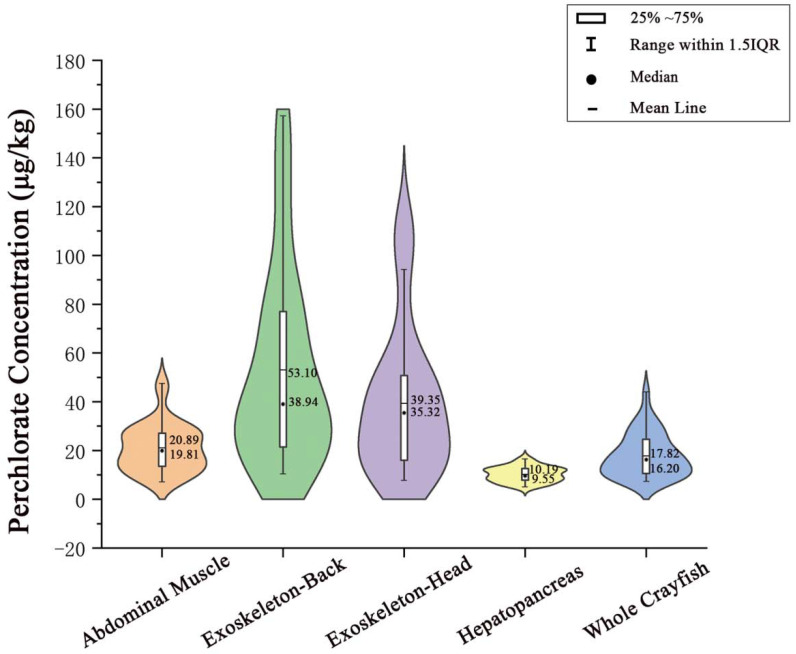
Perchlorate concentration in different tissues of crayfish.

In order to provide an accurate health risk assessment result, it is necessary to obtain the perchlorate contamination levels in the edible parts of crayfish. Therefore, a study on the overview of perchlorate distribution in crayfish was performed. The Violin and Box-whisker diagram presented a wide variation of perchlorate occurrence for different tissues of crayfish (Figure 3). Perchlorate in crayfish tissues displayed significantly different distributions. Our study showed that the highest level of perchlorate occurred in the exoskeleton parts, in which the median concentrations were 38.94 μg/kg for the back and 35.32 μg/kg for the head, respectively, followed by the abdominal muscle at 19.81 μg/kg, and the lowest was in the hepatopancreas, at 9.55 μg/kg. As our results showed in Figure 2, the median value was lower than the mean value in all the types of crayfish tissue, suggesting a positively skewed distribution of perchlorate in these samples. Comparisons of crayfish have been performed for other types of pollutants such as heavy metals, including Cadmium (Cd), Lead (Pb), and Arsenic (As), the results suggested that the digestive organ (hepatopancreas) of crayfish is the site of the highest accumulation for the majority of the studied metals [17,27]. This was inconsistent with our findings on perchlorate. A previous study found that chitosan is a natural adsorbent for perchlorate [28], and the shell of crustaceans is mainly composed of chitin, which may lead to easier interactions between the exoskeleton of crayfish and perchlorate. Then, this combination in turn leads to the highest levels of perchlorate contamination occurring in the exoskeleton of crayfish. Having been compared with the determination result of whole crayfish, it was obvious that perchlorate concentrations in the abdominal muscle mainly corresponded with that of whole crayfish. Pearson correlation analysis was conducted between each kind of tissue with the whole crayfish. The results suggested that a significant correlation only occurred between the abdominal muscle and whole crayfish where the relative coefficient reached 0.378 (*p* < 0.05).

### 3.3. Risk Characterization of Daily Exposure to Perchlorate via Crayfish

**Figure 4:**
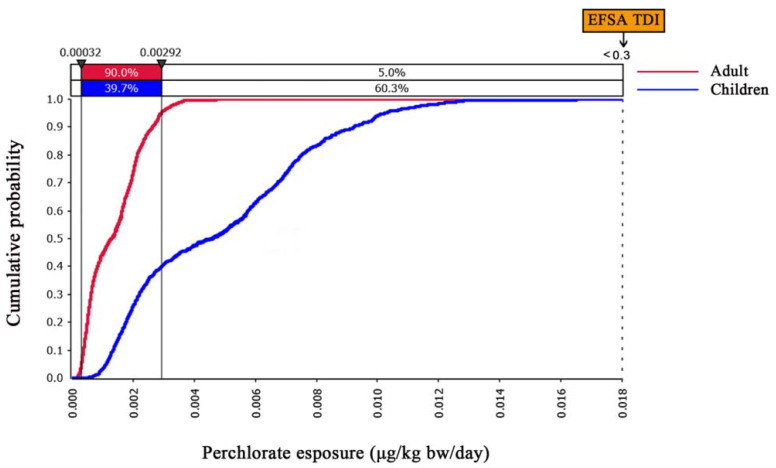
Probabilistic risk characterization of perchlorate exposure in both adults and children. (EFSA: European Food Safety Authority; TDI: Tolerable Daily Intake).

Monte Carlo-simulated risk characterization of EDIs of perchlorate through crayfish are presented in Figure 4. Our results indicated that, for adults, the maximum EDI value of perchlorate induced by ingestion of crayfish was smaller than that for children; similar results have been found in other studies on dietary perchlorate exposure [29]. The curve for the adults showed a faster steady state than the one representing children. The results also suggested that 100% of the EDIs for both the adults and children were below the tolerable daily intake (TDI) value (0.3 μg/kg bw/day) established by the European Food Safety Authority (EFSA) [30]. If the EFSA TDI is used as the threshold for potential risk in the probabilistic assessment, this suggests that crayfish ingestion may indicate a extremely low assessable risk concern for perchlorate exposure in the population.

### 3.4. Probabilistic Assessment of Dietary Perchlorate Exposure from Crayfish

Table 2 summarized the probabilistic approach-estimated daily intake of perchlorate via crayfish (mean, median, and 95th percentage). The results indicated that the mean and P95 values for the dietary intake of perchlorate were 0.0014 and 0.0029 μg/kg bw/day, respectively, for adults, and 0.0049 and 0.0102 μg/kg bw/day, respectively, for children. It was obvious that the EDIs of children were remarkable highly than those of adults. This can be illustrated by the remarkable discrepancy in body weight between the two groups. This was not unexpected considering that children consume more food per body weight; children weigh less than adults, which resulted in higher EDI values [31]. Compared with other results [3,32], the daily intake of perchlorate by ingestion of crayfish was close to that of meats—which were in the same order of magnitude—but significantly lower than the daily intake of perchlorate due to the consumption of vegetables and fruits. In the diet of residents, fruits and vegetables are more essential exposure routes; in addition to the higher perchlorate levels in these foods, dietary habits could be one important factor affecting the results for EDIs. The hazard quotients (HQs) were calculated by dividing the mean EDI values by the RfD of perchlorate (0.7 μg/kg bw/day) across the edible parts—the abdominal muscle and hepatopancreas—of the crayfish. Our results showed that the HQ values were 0.002 and 0.007 for adults and children, respectively, suggesting that children suffered from a greater accumulation risk than adults. Therefore, this factor should be taken into account in the preparation of children’s food. However, the HQ values of both the adults and children were significantly less than one—indicating that perchlorate exposure through the ingestion of crayfish does not pose an appreciable risk to human health.

### 3.5. Sensitivity Analysis of Perchlorate Exposure from Crayfish

A sensitivity analysis could help to identify and rank the factors affecting the assessment outcome. As our results show in Figure S1, a sensitivity analysis of perchlorate exposure from crayfish for both adults and children was performed. The value of the correlation coefficient represents the ability of this variable to influence the average exposure level of perchlorate in crayfish. The results suggest that the correlation coefficient values of perchlorate exposure in the abdominal muscle were 0.39 and 0.38 for adults and children, and 0.16 and 0.20 for perchlorate in the hepatopancreas, respectively. Our findings indicate that the abdominal muscle was much more influential than the hepatopancreas on the average perchlorate concentration in crayfish. The correlation coefficient values for the abdominal muscle were about twice those of the hepatopancreas in the sensitivity analysis for both adults and children. Therefore, the abdominal muscle shows a more important impact on the results of the probability risk assessment.

## 4. Conclusions

In our present study, the occurrence of perchlorate in crayfish obtained from the Middle and Lower Reaches of the Yangtze River was investigated by an acquisition method based on ultra-high performance liquid chromatography coupled with mass spectrometry (UPLC–MS). As the results showed, the range of perchlorate concentrations in cultivated crayfish was 7.74–43.71 μg/kg and 4.90–16.73 μg/kg for market crayfish. It was obvious that the perchlorate level in crayfish obtained from cultivated ponds was higher than that of market crayfish. The distribution of perchlorate in various tissues of crayfish was monitored. The results suggest that the highest perchlorate concentration occurred in the exoskeleton, with a median level of 38.94 μg/kg and 35.32 μg/kg in the back and head, respectively. Monte Carlo simulation was applied to perform a probability risk assessment. The results indicated that the HQ values of both adults and children were less than one, suggesting that there was no appreciable risk to human health from perchlorate via the ingestion of crayfish. This research combined the latest detection technology and analysis methods. We chose crayfish, whose consumption has been increasing in recent years, as the novel research object of this study to investigate its exposure characteristics and to perform health risk assessments for perchlorate, which can provide some new information supporting the formulation of subsequent perchlorate-related national standards.

## Figures and Tables

**Table 1:** Perchlorate levels of crayfish in cultivated ponds and sold in markets (μg/kg).

Source	Region	Mean ± SD	Median	Detection Rate (100%)	Range
Cultivated ponds (*n* = 35)	Xuyi	14.46 ± 4.70	11.96	100	7.74–43.71
	Jianhu	21.76 ± 6.45	25.61	100	
	Huoqiu	29.25 ± 10.19	26.49	100	
	Honghu	10.27 ± 3.78	8.58	100	
	Qianjiang	19.65 ± 8.70	16.47	100	
	Shayang	18.00 ± 8.75	17.52	100	
	Shishou	10.00 ± 3.14	9.42	100	
Markets (*n* = 14)	Nanjing	6.90 ± 1.05	6.90	100	4.90–16.73
	Hefei	10.21 ± 5.86	8.51	100	
	Wuhan	5.17 ± 0.35	5.04	100	
	Changsha	7.79 ± 1.49	8.18	100	
	Chengdu	6.07 ± 0.26	5.93	100	

**Table 2:** Probabilistically estimated daily intake (EDI, μg/kg bw/day) of perchlorate from crayfish.

Edible Parts	Perchlorate Levels (μg/kg)	Adults			Children		
		Mean (SD)	P95	HQ	Mean (SD)	P95	HQ
Hepatopancreas	10.21 ± 3.30	0.0014 (0.0009)	0.0029	0.002	0.0049 (0.0031)	0.0102	0.007
Abdominal muscle	20.98 ± 10.49						

## Data Availability

The data presented in this study are available on request from the corresponding author.

## Supplementary Materials

The following supporting information can be downloaded at: https://www.mdpi.com/article/10.3390/foods11152238/s1, Figure S1: Sensitivity analysis of dietary exposure to perchlorate in crayfish.
