# Supplementary material for: Trends in the Exposure, Distribution, and Health Risk Assessment of Perchlorate among Crayfish in the Middle and Lower Reaches of the Yangtze River
Source: Foods. 2022 Jul 27;11(15):2238. doi: 10.3390/foods11152238 (PMC9368539; doi:10.3390/foods11152238)
Supplement: Supplementary file 1 [file foods-11-02238-s001.zip › foods-1799448-supplementary.pdf]

Supplementary Material For

Trends in the Exposure, Distribution, and Health Risk Assessment of Perchlorate among Crayfish in the Middle and Lower Reaches of the Yangtze River

Mengyuan Chen[1], Manman Wang[1], Bingjie Zhou[1], Mengxin Zhou[1], Qiao Wang[1], Xin Liu[1], Yan Liu[1], Yongning Wu[1,2], Xiaole Zhao[1] and Zhiyong Gong[1,*]

[1] Key Laboratory for Deep Processing of Major Grain and Oil (The Chinese Ministry of Education), Hubei Key Laboratory for Processing and Transformation of Agricultural Products, College of Food Science and Engineering, Wuhan Polytechnic University, Wuhan 430023, China
[2] NHC Key Laboratory of Food Safety Risk Assessment, Food Safety Research Unit (2019RU014) of Chinese Academy of Medical Science, China National Center for Food Safety Risk Assessment, Beijing 100021, China
*Corresponding author: gongzycn@whpu.edu.cn; Tel: +086-27-83924790; Fax: +086-27-83924790

This file includes:

Supplementary Figure

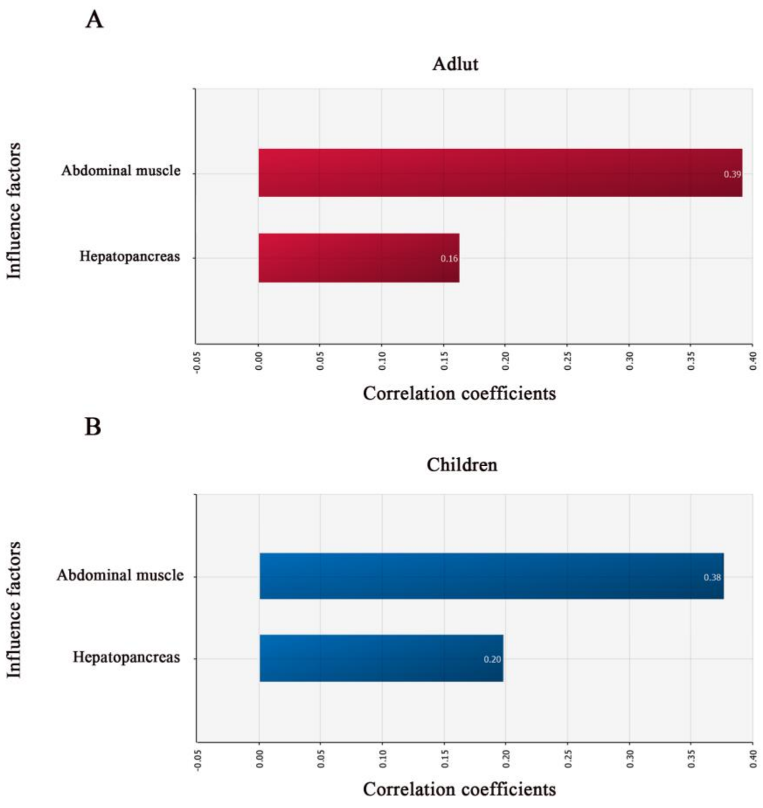

Supplementary Figure S1. Sensitivity analysis of dietary exposure to perchlorate in crayfish.
